# Supplementary material for: Modeling latent infection transmissions through biosocial stochastic dynamics
Source: PLoS One. 2020 Oct 23;15(10):e0241163. doi: 10.1371/journal.pone.0241163 (PMC7584220; doi:10.1371/journal.pone.0241163)
Supplement: S1 File — (PDF) [file pone.0241163.s001.pdf]

S1 File. Program flow

Algorithm 1 Latent Infection Transmissions

INPUT: s_t time series and parameters T_v,T_e, λ_0;
Define object arrays: $Hnode(id,state,t^i,g_{vh},h^i,T_e^i)$, $Vnode(id,state,time,g_v)$, $Edge(id,src,dst,time)$;
Start empty lists *Hexposed, Hactive, Vactive*; add first infected *Hnode* to *Hactive* list; set its parameters as $g_{vh} = 1$, $t^i = 0$,$h^1 = 1$;
Start input time series s_t, which defines time t and temporal resolution; reset counters;
while ($cin >> t >> s_t$) **do**
  in each time step t:
  for all nodes on *Hactive* list **do**
    with prob. $\propto$ the node's susceptibility h^i: create a new *Vnode*; its time is t, and its $g_v = g_{vh}$ is transfered from the creator *Hnode*, the state is "infectious"; put it to *Vactive* list; create an Edge from *Hnode*$\rightarrow$ *Vnode*, specify its time as t;
  end for
  Update the transmission probability λ_t (see text);
  for all $1 \leq i \leq s_t$ **do**
    Create a new *Hnode* and set its properties: state="uninfected"; susceptibility h^i as rand$\in [0,1]$; its exposure T_e^i as rand $\in [1,T_e]$, and set $g_{vh} = 0$. Add the node to *Hexposed* list;
  end for
  for all nodes on *Hexposed* list **do**
    with prob. λ_t (modified by the node's h^i and $g(g_v)$) connect the *Hnode* to a random node on *Vactive* list; change its state to "infected" and add to the *Hactive* list; update $g_{vh} = g_v + 1$ from that *Vnode*; create the Edge from *Vnode*$\rightarrow$*Hnode*; mark its time as t;
  end for
  Revise the lists *Vactive*, *Hexposed* and *Hactive* regarding the respective difference Δt between the current time t nd the node's time;
  for all nodes in *Vactive* list **do**
    if $\Delta t > T_v$ **then**
      (virus survival time exceeded): remove from the list;
    end if
  end for
  for all nodes in *Hexposed* list **do**
    if the status changed to "infected" **then**
      (infected): remove from the list;
    else
      if $\Delta t > T_e^i$ **then**
        (the node's exposure time exceeded): remove from the list;
      end if
    end if
  end for
  for all nodes in *Hactive* list **do**
    if $\Delta t > 14$ days **then**
      (recovered): remove from *Hactive* list;
    else
      if the node's $h^i > 0.8$ **then**
        compute T_h as rand $\in [2,7]$ days;
        if $\Delta t > T_h$ **then**
          (hospitalized): remove from the list;
        end if
      else
        keep on *Hactive* list;
      end if
    end if
  end for
  Sampling temporal quantities of interest;
end while
Sampling network and statistical quantities of interest;
END
