# Supplementary material for: Modeling latent infection transmissions through biosocial stochastic dynamics
Source: PLoS One. 2020 Oct 23;15(10):e0241163. doi: 10.1371/journal.pone.0241163 (PMC7584220; doi:10.1371/journal.pone.0241163)
Supplement: Attachment — Submitted filename: response_n.pdf [file pone.0241163.s003.pdf]

5**. Review Comments to the Author**

Please use the space provided to explain your answers to the questions above. You may also include additional comments for the author, including concerns about dual publication, research ethics, or publication ethics. (Please upload your review as an attachment if it exceeds 20,000 characters)

Reviewer #1: This work develop an agent-based model for simulations of infection transmission in an open system driven by the dynamics of social activity; the model takes into account the personal characteristics of individuals, as well as the survival time of the virus and its potential mutations. The paper is well organized. It can be seen that the proposed model reveal complex feedback mechanisms that shape the dependence of the infection curve on the intensity of social dynamics and other sociobiological factors.

Authors response:
We appreciate the Reviewer's time and a deep understanding of our work.

Reviewer #2: Thank you for the opportunity to read this interesting manuscript. The manuscript is very easy to read. Authors clearly describe their aims, provide clear operationalization of all variables, and the reasoning of the procedure. In general, I consider this type of simulation to have a contribution over the mean-field theory whose results are biased by the well-mixed assumption and missing the temporal dynamics in real-world epidemic spreading.

Authors response:
We thank the Reviewer for his/her time and for very encouraging statements about our work.

The authors propose an agent-based model to address the latent infection transmissions by introducing temporal dynamics in simulation. The most impressive and practical thing is the authors treat the human and virus as separate elements in their model. The introduction of exposure time for the human and survival time for the virus can also reflect the latent characteristic of infection transmissions. Besides, the application of parameter gv helps capture the weakening process of the virus due to evolution.

The authors used the data collected from online social networks, MySpace, as the input data regarding human dynamical fluctuations over time. *However, the disease spreading in the real-world depends on contacts (either face-to-face contacts or indirect face-to-face contacts), which may significantly distinct from online social interactions between people.* Rather than data from online social media, I miss a modification of this model using data concerning direct or indirect face-to-face contacts. Or, perhaps, the authors could provide a more solid justification for their using of the online social interactions in the context of infection transmission.

Authors response:
We thank the Reviewer for pointing out these issues, which, indeed, need to be better clarified, as we do in the revised text. Specifically, in the absence of real-life data sets, we use a first proxy of the social dynamics available. Among the online social networks, the MySpace data sets are such that they most closely reflect the off-line community contacts and their mutually triggered activity, as our previous studies in Refs.[8,46] show. Moreover, these data sets contain temporal correlations (persistent fluctuations that precisely match the off-line activity fluctuations) and circadian cycles, both of which define essential features of human activity patterns. We added a brief explanation in favour of this empirical time series, as compared to the alternatives (a computer-generated time series or a random number).

We have expanded the Discussion section to once again stress the absence of geographical location and mobility patterns of the agents, also referring to different approaches in Ref.[35,41,54], where a concrete data of such kind are collected and used within the model, aiming to predict the impact of mobility that provides contacts and transmission among different groups of the population.

Furthermore, we pointed out the limitations of our model as well as possibilities to expand the properties of the agents. For example, the model can take into account the heterogeneity in the

number of contacts along which the virus could be transmitted, possibly with a changed rate, as well as temporal variations of the viral load, as some recent empirical data indicate. These issues remain to be studied in future work.

As a more specific remark, I would have expected clarification (or supporting materials) of this assumption: ‚thus, more susceptible agents, who are likely to have severe symptoms, can spread viruses more often than those who are barely ill.‚Äù I miss a brief description of the schematic of how virus nodes infect human nodes, or which virus node infects which human node. I also miss I miss a more robust discussion on the limitations of the model.

Authors response:

We thank the Reviewer for pointing out this issue. Different infectiousness of the agents, especially between asymptomatic and symptomatic individuals, needs to be clarified. Recent studies, for example, in Refs. [47,48,55], found that the viral load varies during the infectious time and that the average viral load in the upper respiratory tract of mild and severe symptomatic individuals is proportionally different. Also, a summary in Ref.[18] points out that "80,9% were considered asymptomatic or mild pneumonia but released large amount of viruses at the early phase of infection". We do not have enough data to adequately introduce another variable for the temporal variations of the viral load of each agent; therefore, we use the susceptibility h^i that effectively differentiates asymptomatic from symptomatic ones. In the Model description, we added a comment regarding different viral loads that motivates the corresponding model rule. Furthermore, we mentioned the occurrence of the temporal patterns of viral loads in the Discussion by pointing out the limitations and potential extension of our model.

In the graph, we have precisely the link along which one host infects the other one via its virus. Perhaps, the Reviewer is pointing to potential (social) relationships between the hosts, which we do not take into account in this version of the model, as above described. Thus, we randomly select among currently existing active viruses; each of them is posted by a known active carrier. In the expanded Discussion, we pointed out several limitations of the current version of the model, as stated above.

%%%
